# Effect of Formulation and Process Variables on the Stability and Quality Attributes of Digoxin Formulations

**DOI:** 10.3390/ph19081185

**Published:** 2026-07-29

**Authors:** Rizwan Shaikh, Bhanu P. Dongala, Swaroop J. Pansare, Sunil K. Thota, Mohammad T. H. Nutan, Theresa U. Offili, Mansoor A. Khan, Ziyaur Rahman

**Affiliations:** 1 Irma Lerma Rangel College of Pharmacy, Texas A&M Health Science Center, Texas A&M University, College Station, TX 77843-1114, USA; rizwansk@tamu.edu (R.S.); bhanu.dongala@tamu.edu (B.P.D.); swaroop0205@tamu.edu (S.J.P.); sunilkthota@tamu.edu (S.K.T.); tofili@tamu.edu (T.U.O.); mkhan@tamu.edu (M.A.K.); 2 Irma Lerma Rangel College of Pharmacy, Texas A&M Health Science Center, Texas A&M University, Kingsville, TX 78363-8202, USA; mnutan@tamu.edu

**Keywords:** narrow therapeutic index, particle size, digoxin, crystallinity, dissolution, stability

## Abstract

**Background:** Digoxin (DGX) is a narrow therapeutic index (NTI) and Biopharmaceutics Classification System (BCS) class IV drug whose clinical performance can be influenced by physicochemical properties, formulation composition, manufacturing processes, and stability-related solid-state transformations. **Objective:** The objective of the paper was to understand the effect of process and formulation variables on digoxin formulations. **Methods:** This study reports the effect of particle size, lactose types (LA and LM), manufacturing methods, and granulating fluid (ethanol and water–ethanol) on the critical quality attributes of DGX tablets (XRPD, DT, assay, impurity, and dissolution). Accordingly, 10 formulations were prepared using a full factorial design and monitored for 30 days at 40 °C/75% RH. **Results:** Assay values of the formulations did not change significantly (*p* > 0.05) compared to initial values after storage. No impurities or new peaks were detected in the stability samples, indicating either no drug degradation or the degradation products were below the limit of detection of the method (31.5–44.6 ng/mL). The dissolution rate and extent of the formulations changed after exposure, indicating the effect of composition and process variables. In direct compression formulations, a decrease in dissolution rate, especially at 10 and 20 min, was higher than at 60 min, which may be related to an increase in hardness and DT values. However, a decrease in dissolution of 5.6–8.7% at 60 min was observed after storage from the initial values, which may be due to an increase in crystallinity. **Conclusions:** Particle size, manufacturing method, and granulating fluid significantly influenced the solid-state characteristics and dissolution performance of DGX tablets. Wet granulation promoted partial amorphization and improved initial dissolution but reduced stability due to recrystallization during storage. These findings highlight the importance of formulation and process selection in ensuring the quality and stability of NTI drug products.

## 1. Introduction

The biopharmaceutical classification system (BCS) is a scientific framework that classifies drug substances based on their aqueous solubility and intestinal permeability into four categories: class I (high solubility and high permeability), class II (low solubility and high permeability), class III (high solubility and low permeability), and class IV (low solubility and low permeability) [1]. BCS class IV drug is known for clinical variability, which arises from variability in dissolution, permeability, and bioavailability [2,3]. Furthermore, this class of drugs poses challenges in developing formulations with consistent and robust quality attributes, which ensure consistency in clinical outcome. One of the BCS class IV drugs is digoxin (DGX), and it is used to control the symptoms of congestive heart failure. The clinical outcome of DGX may be affected by the product quality, which is linked to its physicochemical properties [4,5]. Examples of DGX physicochemical properties are particle size, polymorphism, solubility, dissolution, permeability, etc. Besides physicochemical properties, excipients, manufacturing methods, process variables, and stability are expected to have a significant impact on its product quality.

Particle size is known to affect dissolution rate, which in turn impacts the bioavailability of drugs [6,7,8,9,10,11]. Literature has reported that the reduction in particle size of DGX leads to an increase in both the rate and extent of drug absorption [12,13]. Particle size is a key determinant in bioavailability because it may affect the dissolution rate [14,15]. Similarly, amorphous and crystalline forms of the drug have a significant impact on dissolution rate and bioavailability. DGX exists in various polymorphic forms, including hydrate (hemihydrate), anhydrous, and amorphous [16]. The anhydrous forms of DGX dissolve faster, while hydrate forms dissolve slowly [17]. Previous studies have shown that the amorphous form of the drug has better compressibility, higher solubilization, and dissolution rate than its crystalline form. Amorphous forms are thermodynamically unstable and may revert to a stable crystalline form. This is manifested in decreased dissolution and bioavailability [18,19].

Quality of a drug product can be modulated by excipient and manufacturing method [20,21,22]. It is critical to understand the interplay of the drug physicochemical properties, excipients, and manufacturing methods that influence DGX quality. Furthermore, the literature has reported a significant impact of excipient, manufacturing method, and process variables on product quality, especially NTI drugs such as warfarin, phenytoin sodium, tacrolimus, etc. For example, Phenytoin sodium has shown its quality to be affected by excipients such as lactose type, manufacturing method, and process variables, leading to a decrease in dissolution due to phase transformation happening during the manufacturing process. Similarly, the formulation, manufacturing process, and stability conditions have a significant influence on the quality of warfarin sodium products. It is expected that excipients, physicochemical properties, manufacturing methods, and process variables may have a significant impact on quality. Based on this assumption, this study was undertaken. The first objective of this study was to understand the interplay between physicochemical properties (particle size), excipients (lactose type) and manufacturing methods (dry and wet granulation) on the critical quality attributes of DGX tablets. The second objective of this study was to assess the impact of short-term stability exposure on product quality. To the best of our knowledge, this type of study on DGX has not been reported in the literature.

## 2. Results and Discussion

### 2.1. Initial Characterization of Formulations

#### 2.1.1. X-Ray Powder Diffraction

XRPD of DGX exhibited characteristic reflection bands at 13.5, 15.2, 16.8, 17.5, and 18.5° indicating the crystalline nature of the drug. The placebo mixtures showed characteristic reflection bands of LM or LA. On the other hand, PM-1 and PM-2 did not show all the reflection bands for the drug except the reflection band at 16.8° due to a low percentage of drug relative to the excipients of the matrix. A similar diffractogram was observed in the formulations. However, the reflection band of the drug appeared with varying intensity, indicating the effect of formulation and process variables on the solid-state of the drug. To assess the effect of formulation and process variables on the solid-state of the drug (crystallinity), reflection band intensity and raw area at 16.8° were monitored and compared to PM to measure the relative crystallinity. This approach provided a relative measure of the drug crystallinity before and after processing under the various experimental conditions. The analysis was based on the diffraction peak with the highest intensity that was free from interference by the formulation excipients. Furthermore, XRPD was the only analytical technique capable of detecting the drug-specific diffraction peak because of the extremely low drug dose. Other techniques, including DSC, Raman spectroscopy, and FTIR spectroscopy, were unable to reliably detect or characterize the drug in the formulations due to its low concentration. The influence of formulation variables on crystallinity (Y_1_) was represented by the regression equation:

$$Crystallinity\;\left(Y_{1}\right)=46.992X_{0}-0.275X_{1}+1.967X_{2}+1.133X_{3}+1.9825X_{1}X_{2}+3.6475X_{1}X_{3}-1.74525X_{3}$$

The regression model further supported the XRPD observations by describing the relative contribution of the formulation variables to drug crystallinity. Although the individual model terms were not statistically significant, the model showed excellent agreement with the experimental data (R^2^ = 0.9928; RMSE = 1.2587). Among the interaction terms, lactose type–granulating solvent (X_1_X_3_) exhibited the largest positive coefficient (+3.647), indicating that the influence of the granulating solvent on crystallinity was strongly dependent on the lactose grade employed. Particle size (+1.967) and granulating solvent (+1.133) also showed positive contributions to crystallinity, whereas lactose type alone (−0.275) exerted only a minimal effect. These model predictions were consistent with the experimental XRPD results discussed below.

**Figure 1:**
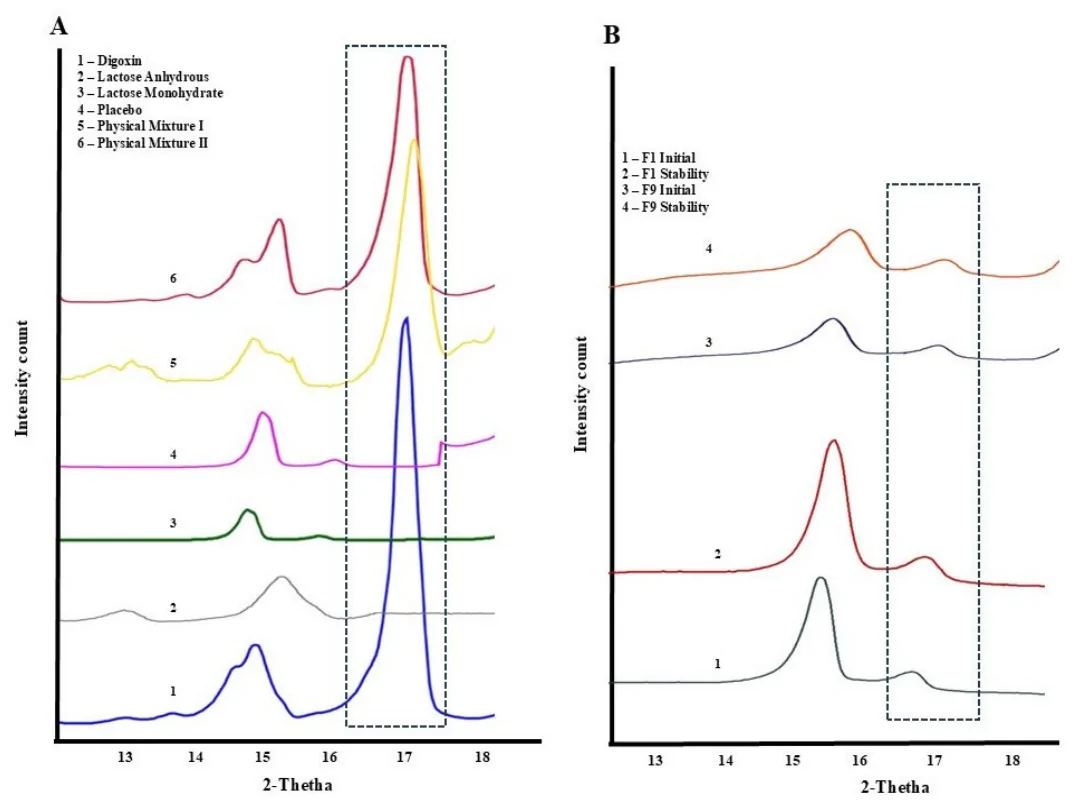
Diffractograms of (**A**) digoxin, lactose anhydrous, lactose monohydrate, placebo, and physical mixtures; (**B**) selected digoxin formulations (F1 and F9) before and after storage at 40 °C/75% RH, indicating changes in crystallinity.

Directly compressed formulations showed higher intensity and raw area compared to the wet-granulated ones. For instance, band intensity in the wet-granulated F5 formulation (2.45 × 10^5^ counts) was almost 2-fold less than that of the physical mixture F10 formulation (4.95 × 10^5^ counts) that was prepared by direct compression. The corresponding decrease in crystallinity of the F5 formulation was almost 43.4% by comparing the raw area of the band, and the physical mixture F10 was used as the relative crystalline reference (100% relative crystallinity). This may be due to the crystalline-to-amorphous phase transformation of the drug, which was mediated by the granulating fluid [23]. Some fraction of the drug may dissolve in the solvent during granulation, resulting in reduced crystallinity, consistent with the positive contribution of the granulating solvent (X_3_, +1.155) in the regression model. Furthermore, the type of granulation fluid had an impact on the extent of solid-phase transformation. Formulations granulated with water–ethanol showed a higher degree of decrease in crystallinity compared to ethanol-granulated ones. For example, the ethanol-granulated formulation (F2) showed higher intensity (3.86 × 10^5^ counts) than the ethanol-water granulated F8 formulation. A decrease in crystallinity was 47.9 and 63.5% in F2 and F8, respectively. This can be explained by the solubility characteristics of the DGX in granulating solvents. Approximate solubility of the drug was determined in ethanol and a water–ethanol mixture by adding known quantities at 25 °C and found to be 1.53 mg/mL and 2.51 mg/mL, respectively. During granulation, a greater fraction of the drug may solubilize in water–ethanol compared to ethanol alone. Upon drying, the solubilized drug may dissolve with the binder and convert into an amorphous form but not into a crystalline form due to the fact that crystallization is a time-dependent phenomenon. Moreover, an amorphous drug is thermodynamically unstable and may revert to the crystalline form at higher temperatures and humidity [24]. Particle size had an impact on the amorphous phase content formed during granulations. Smaller particles formed higher amorphous content than larger particle sizes due to a higher dissolution rate in the solvent during granulation. For instance, F3 (75 µm) and F8 (32 µm) contained 48.0 and 35.8% crystalline fraction relative to physical mixture F10 (Figure 1), in agreement with the positive coefficient for particle size (X_2_, +1.967), which identified particle size as the dominant factor influencing crystallinity.

#### 2.1.2. Disintegration Time

**Figure 2:**
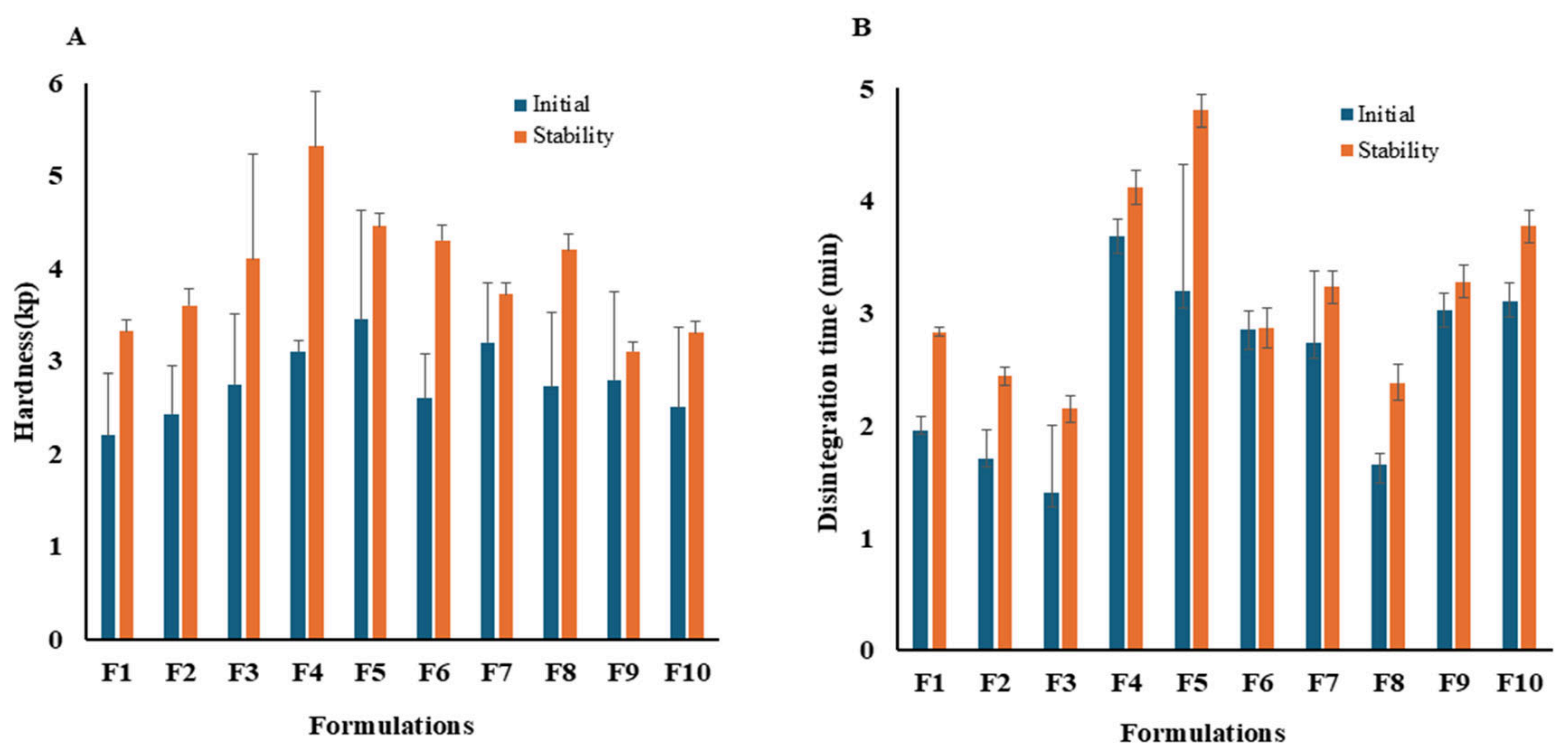
(**A**) Tablet hardness and (**B**) disintegration time of digoxin formulations before and after storage at 40 °C/75% RH.

Disintegration time (DT) varied from 1.40 ± 0.3 (F3) to 3.68 ± 0.60 (F4) min depending on the various factors selected for formulations (Figure 2). All studied factors (particle size, lactose type, and granulating fluid) had a statistically insignificant effect (*p* > 0.05) on the DT of the tablets. The influence of formulation variables on DT was described by the following regression equation:

$$DT\;\left(Y_{2}\right)=3.076X_{0}+2.839X_{1}+1.339X_{2}+0.091X_{3}+1.356X_{1}X_{2}+0.098X_{1}X_{3}-0.916X_{2}X_{3}$$

The regression model adequately described the experimental DT response (R^2^ = 0.9400; RMSE = 2.4996) and provided additional insight into the relative influence of the formulation variables on tablet disintegration. Although the individual model terms were not statistically significant, the comparatively larger lactose type–particle size interaction coefficient (+1.356) suggests that these variables collectively influenced tablet disintegration, complementing the experimental observations discussed below. Among the evaluated factors, lactose type had the greatest influence on DT, which is consistent with the regression model where lactose type exhibited the largest positive coefficient (+2.839), followed by particle size (+1.339), while the granulating solvent showed only a minimal contribution (+0.091). LA demonstrated a stronger effect on DT than LM. This observation can be explained by differences in lactose solubility. LM has lower solubility due to the crystallization of water within its crystal lattice, which limits the wetting of the internal part of the powder. In contrast, LA lacks lattice-bound water, which allows water to penetrate more rapidly and results in faster tablet breakup, resulting in shorter DT [25]. A similar trend was observed in F1 and F8 containing LM and LA, with DT values of 1.96 ± 0.4 min and 1.65 ± 0.7 min, respectively, consistent with the reported literature [26]. In addition to solubility differences, LA (SuperTab^®^ 22AN) exhibited a smaller particle size (~20–30 µm) compared to LM (SuperTab^®^ 14 SD), which had a particle size (~130–150 µm); the LA provided a higher surface area for water penetration, contributing to faster DT [27,28]. Although the granulating fluid showed minimal overall impact on DT, as reflected by its small regression coefficient (+0.091), F5 showed slightly longer DT (1.98 ± 0.7 min) compared to F2 (1.71 ± 0.3 min), despite both formulations containing LM and the same drug particle size. This difference may be attributed to enhanced granule densification associated with LM, indicating greater LM solubility in the water–ethanol (50:50, *v*/*v*) mixture than in ethanol alone, resulting in denser granules. In particular, the longer DT observed for the wet-granulated formulation may be due to stronger interparticle bonding formed during granulation in the presence of granulating fluid (F4: 3.68 ± 0.5 min). In contrast, the direct compression formulation F10 likely retained greater internal porosity, resulting in faster water penetration into the tablet and a comparatively shorter DT (3.11 ± 0.9 min) [29]. Furthermore, the relatively large positive interaction coefficient between lactose type and particle size (+1.356) suggests that these variables collectively influenced water penetration and tablet breakup to a greater extent than either factor alone, consistent with the experimentally observed differences in DT among the investigated formulations.

#### 2.1.3. Assay and Impurities

This process variable had a statistically insignificant impact (*p* > 0.05) on assay and impurities. According to the USP monograph of the DGX tablet, it should contain not less than 90% and not more than 105% of the labeled amount of the drug. All formulations complied with the assay specification of the monograph. Assay limits varied from 99.4 ± 1.1 (F1) to 102.1 ± 1.3% (F9), respectively. Moreover, content uniformity was not evaluated because of the small batch size (~50 tablets). However, content uniformity testing should be performed for commercial-scale batches to ensure dose uniformity and compliance with pharmacopeial requirements. DGX is a cardiac glycoside, and it is prone to hydrolytic degradation into its sugar (glycone) and non-sugar (aglycone) components. Three major impurities (DGB, DGM, and DGG) are typically formed during hydrolysis, all of which retain the alpha, beta-unsaturated lactone ring and the C14 (β)-hydroxyl group, both critical to the drug’s pharmacological activity [30]. No detectable impurities were observed across all formulations.

#### 2.1.4. Dissolution

The USP monograph describes two dissolution methods with different specifications for DGX tablets. The first method recommends 500 mL of 0.1 N hydrochloric acid as the dissolution medium, with a minimum dissolution of 85% within 60 min [31]. The second method specifies 500 mL of water as the dissolution medium, with a minimum dissolution of 85% within 20 min. Analysis of dissolution data demonstrated that particle size and granulating solvent were statistically significant main factors affecting early-stage dissolution at T_10_ (Y_4_, dissolution at 10 min) and T_30_ (Y_5_, dissolution at 30 min) (*p* < 0.05). The regression models describing dissolution at T10, T30, and T60 (Y_5_, dissolution at 60 min) were:

$$Dissolution\;at\;T_{10}\;\left(Y_{3}\right)\;=\;11.187X_{0}\;-\;0.362X_{1}\;-\;6.362X_{2}\;-\;3.063X_{3}\;-\;0.412X_{1}X_{2}\;+\;0.887X_{1}X_{3}\;+\;2.237X_{2}X_{3}$$

$$Dissolution\;at\;T_{30}\;\left(Y_{4}\right)\;=38.057X_{0}-1.200X_{1}-21.800X_{2}-10.725X_{3}-1.400X_{1}X_{2}+3.925X_{1}X_{3}+9.625X_{2}X_{3}$$

$$Dissolution\;at\;T_{60}\;\left(Y_{5}\right)\;=58.250X_{0}-1.900X_{1}-22.520X_{2}-9.000X_{3}+0.375X_{1}X_{2}-0.050X_{1}X_{3}+10.075X_{2}X_{3}$$

The regression models demonstrated a very close fit with the experimental dissolution data, with high R^2^ values and low RMSEs (dissolution at T_10_: R^2^ = 0.9973, RMSE = 1.096; dissolution at T_30_: R^2^ = 0.9923, RMSE = 1.2799; dissolution at T_60_: R^2^ = 0.9919, RMSE = 6.7175). At T_10_, particle size was the only statistically significant factor and exhibited the largest negative coefficient (−6.362), indicating that increasing particle size reduced the amount of drug released during the initial stage of dissolution. At T_30_, both particle size (−21.800) and granulating solvent (−10.725) significantly influenced drug release, while the positive interaction coefficient (+9.625) indicates that the influence of particle size on dissolution depended on the granulating solvent employed.

**Figure 3:**
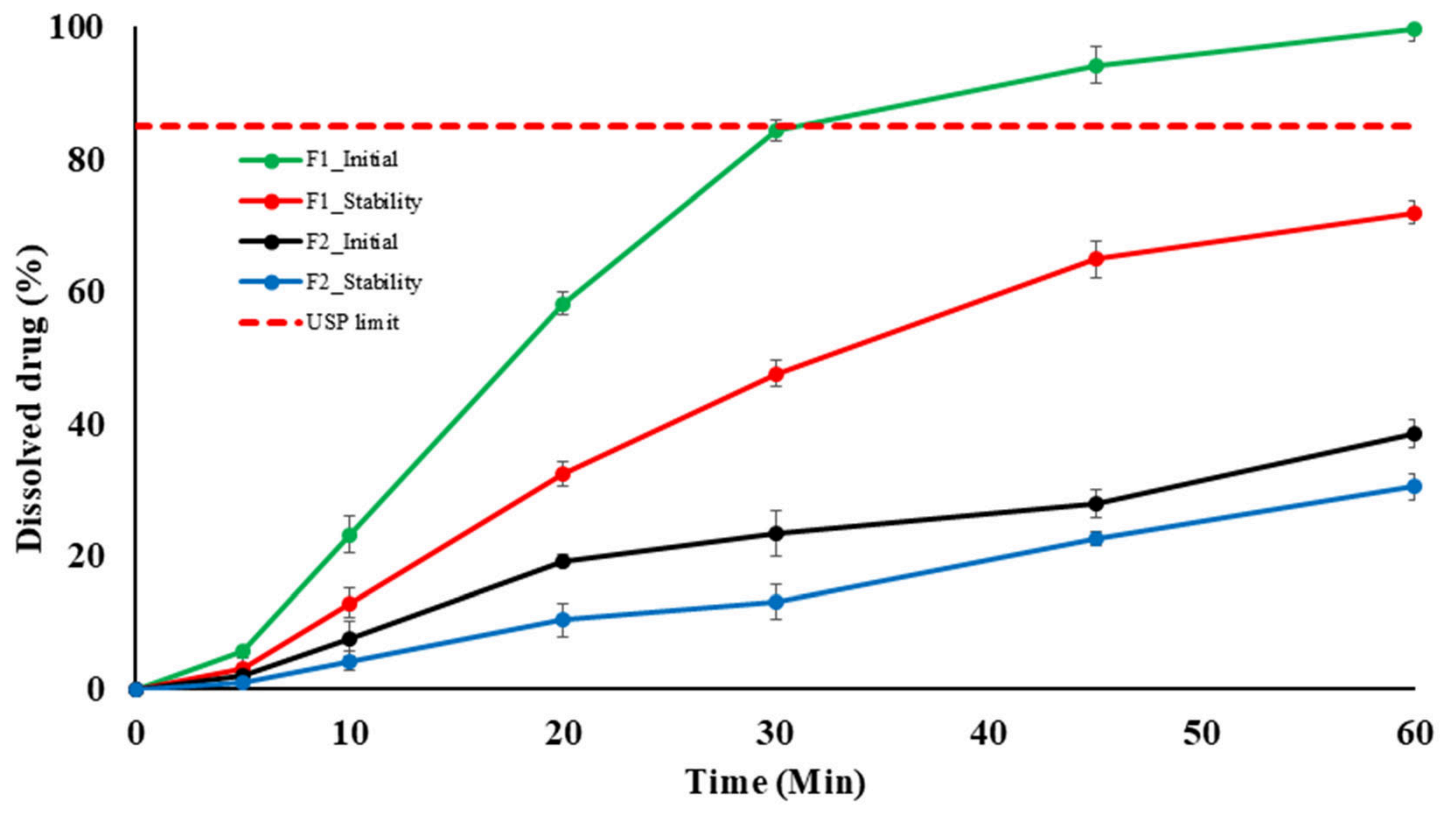
Dissolution profiles of formulations F1 and F2 before and after storage at 40 °C/75% RH.

**Figure 4:**
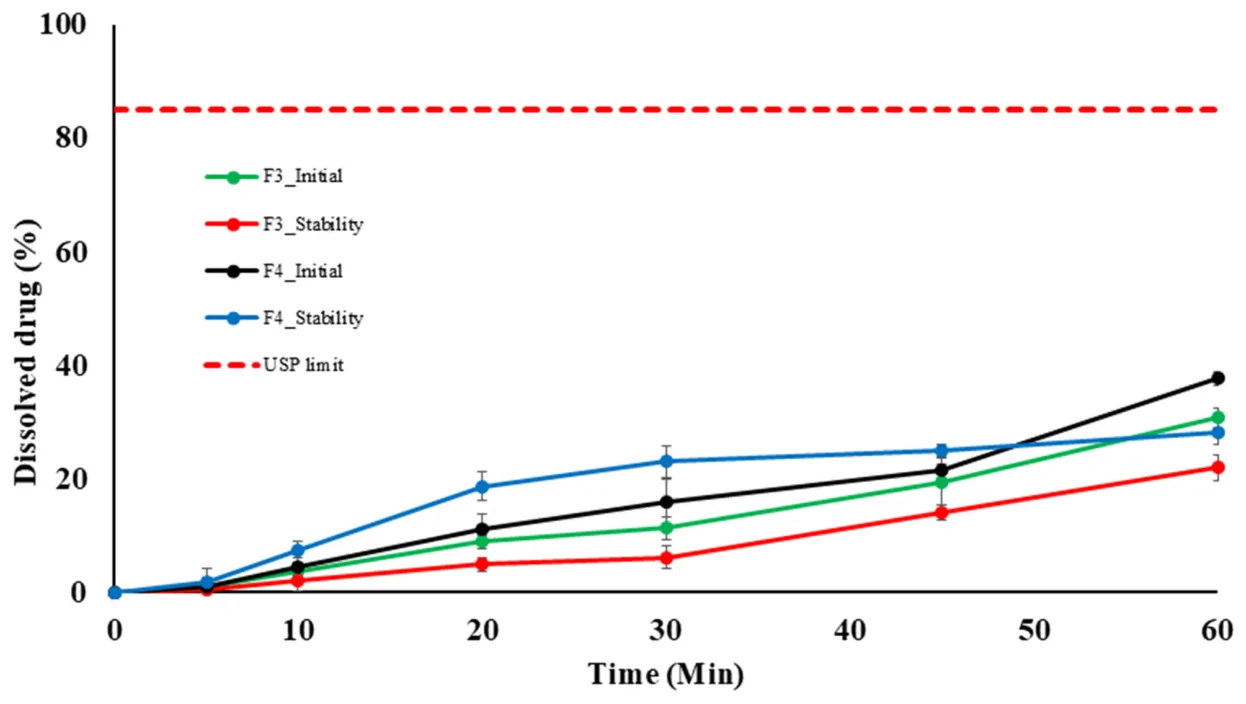
Dissolution profiles of formulations F3 and F4 before and after storage at 40 °C/75% RH.

**Figure 5:**
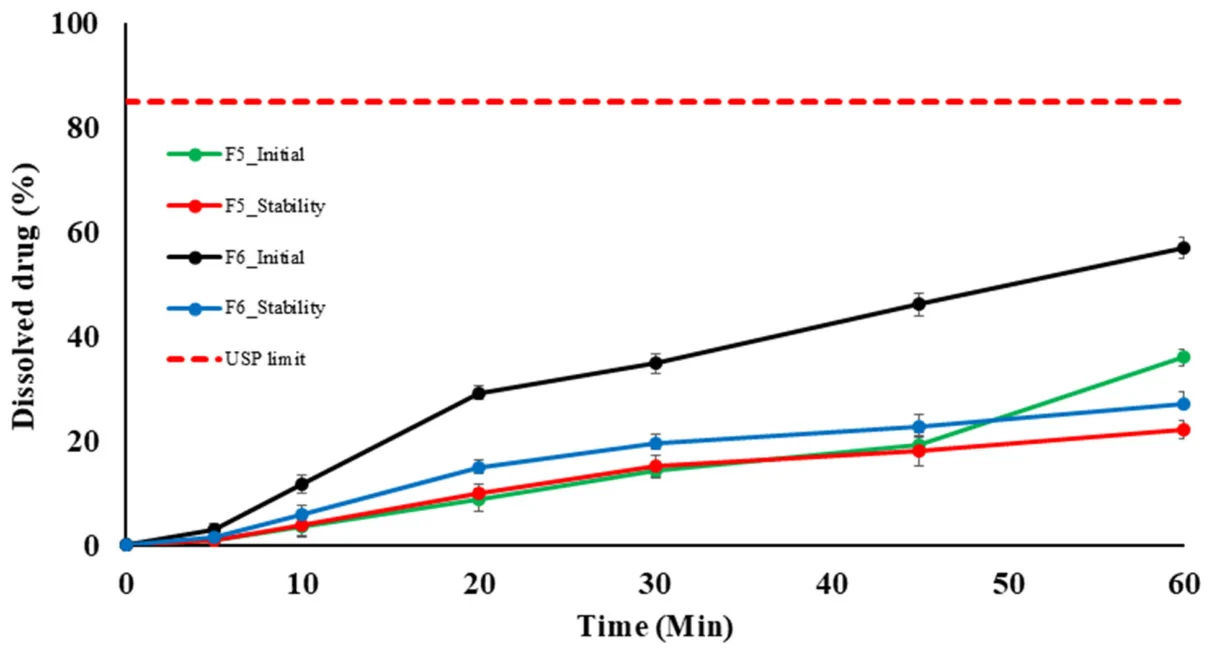
Dissolution profiles of formulations F5 and F6 before and after storage at 40 °C/75% RH.

**Figure 6:**
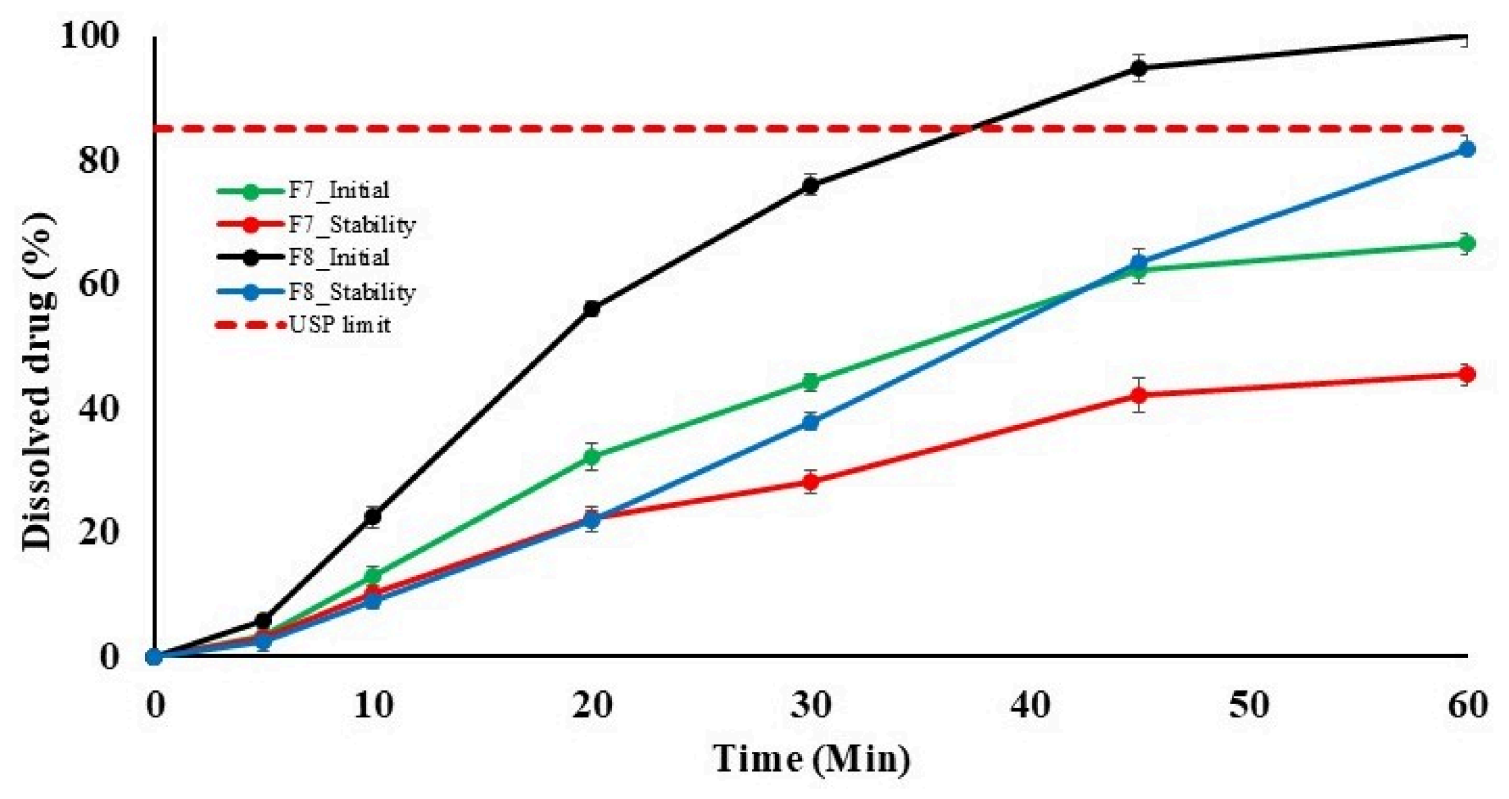
Dissolution profiles of formulations F7 and F8 before and after storage at 40 °C/75% RH.

**Figure 7:**
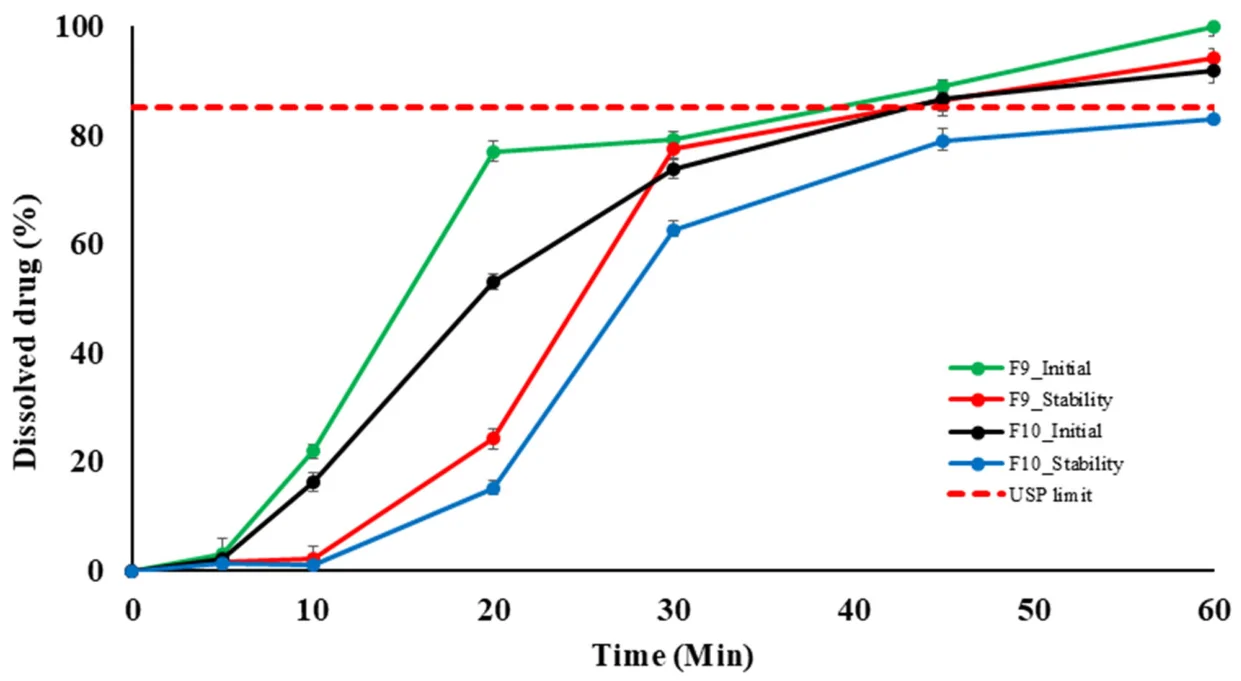
Dissolution profiles of formulations F9 and F10 before and after storage at 40 °C/75% RH.

The drug particle size (D_90_) showed a clear influence on dissolution in both directly compressed and wet-granulated formulations. Specifically, formulations containing a smaller drug particle size (32 μm) produced a higher dissolution rate and extent compared to formulations prepared with a larger drug particle size (75 μm) (Figure 3, Figure 4, Figure 5, Figure 6 and Figure 7). This trend can be explained by the Noyes–Whitney equation [32,33]. A reduction in particle size is accompanied by an increase in the surface area available for dissolution, consequently increasing the dissolution rate [32,34,35]. Dissolution profiles of directly compressed formulations did not differ significantly compared to wet-granulated formulations. Directly compressed formulations F9 (32 μm) and F10 (75 μm), having identical compositions, showed an insignificant effect of particle size and dissolved 99.9 ± 1.8% and 91.8 ± 1.3% drug in 60 min, respectively. On the other hand, F3 and F8 formulations showed statistically significant (*p* < 0.05) differences in dissolved drug at 60 min (Figure 4 and Figure 6). This was related to the granulation process and entrapment of the dissolved drug in the binder matrix since both drug and binder are soluble in the granulation fluid. Dissolved drug was 30.8 ± 1.7 and 100.8 ± 1.3% in 60 min in F3 and F8, respectively. F8 showed higher dissolution than F3 due to a higher proportion of the amorphous fraction and smaller particle size, as supported by the XRPD data. This observation is consistent with the positive particle size–granulating solvent interaction coefficients in the regression models (+2.237, +9.625, and +10.075 for T_10_, T_30_, and T_60_, respectively), suggesting that the effect of particle size on drug release became increasingly dependent on the granulation solvent throughout the dissolution process. Lactose type appeared to have little influence on dissolution across formulations prepared by different methods or particle sizes, consistent with its comparatively small regression coefficients (−0.362, −1.200, and −1.900 for T10, T30, and T_60_, respectively).

### 2.2. Stability Study

Assay values of the formulations did not change significantly (*p* > 0.05) compared to initial values after storage. The formulations remained within the acceptance criteria as defined in the USP monograph of the DGX tablet (95–105%). Initial assay ranged from 99.4 ± 1.1 (F1) to 102.1 ± 1.3%, while after stability, it ranged from 97.1 ± 1.6 to 100.1 ± 1.3%. No impurities or new peaks were detected in the stability samples, indicating either no drug degradation or that the degradation products were below the limit of detection of the method (31.5–44.6 ng/mL). Significant changes were observed in the hardness, DT, dissolution, and solid-state after storage. The tablet hardness was increased in both direct compression and wet granulation formulations, although the increase was more pronounced in wet granulated formulations. For example, hardness increased from 2.8 ± 0.3 to 3.1 ± 0.4 kp in the direct compression (F9), whereas in the wet granulated formulation (F7) it increased from 3.2 ± 0.8 to 3.7 ± 0.2 kp, representing an approximately 1.2-fold increase. A statistically significant (*p* < 0.05) increase in DT was observed in all formulations relative to the initial values, and the increase was approximately 1.1–1.7-fold from their initial values. Direct compression formulations showed an increase in DT from 3.02 ± 0.9–3.11 ± 0.6 to 3.28 ± 0.9–3.77 ± 0.8 min after storage. Although the granulating fluid showed an impact on DT, the F5 formulation showed approximately a 12% increase in DT compared to F2. An increase in hardness and DT may be attributed to physical and microstructural modifications within the tablet mediated by weight gain and humidity. Microstructural modifications related to bonding formation between binder and matrix components [29,36]. Directly compressed formulations contain HPC as a binder in particulate forms that swell to some degree on exposure to humidity [37,38,39]. The swollen binder spread and formed bonds with the matrix of the tablets, resulting in an increase in mechanical strength and DT. Similarly, wet granulated formulations showed a higher increase in DT compared to directly compressed formulations. Wet granulation formulations showed DT change from 1.40 ± 1.8–3.68 ± 1.3 to 2.15 ± 1.1–4.81 ± 0.7 min after stability exposure. Hardness and DT behavior differences between directly compressed and wet granulated formulations can be explained by degree of weight gain and microstructure changes in the matrix. In the wet granulated formulation, some fraction of the binder was already present as a thin layer, spread onto the excipients. On humidity exposure, higher weight gain and swelling resulted in higher spread onto excipients, and bonding that resulted in an increase in mechanical strength and DT. Direct compressed formulations showed approximately an increase in weight gain of 2% compared to 5% in wet granulated formulations [40,41].

The dissolution rate and extent of the formulations changed after exposure, indicating the effect of composition and process variables (Figure 5 and Figure 6). In direct compression formulations, the decrease in dissolution rate, especially at 10 and 20 min, was higher than at 60 min, which may be related to an increase in hardness and DT values. However, a decrease in dissolution of 5.6–8.7% at 60 min was observed after storage from the initial values, which may be due to an increase in crystallinity. An increase in crystallinity of 11.9–17.2% was observed from the initial values by comparing the raw area of the reflection band at 16.8°. Dissolution of F9 and F10 formulations decreased from 99.9 ± 1.1 and 91.8 ± 0.9 to 94.3 ± 1.6 and 83.1 ± 2.1%, respectively (Figure 7). It is possible that some amorphous form of the drug was generated during the compression step of the manufacturing process that converted to a crystalline form during the stability period, as amorphous form of the drug is thermodynamically unstable [42,43,44]. A more pronounced decrease in dissolution rate and extent was observed in wet-granulated formulations compared to directly compressed formulations after storage. This may be due to an increased crystallinity after storage, as the initial crystallinity of wet-granulated formulations was relatively low. For instance, formulation F8 showed a significant decrease in both rate and extent compared to F9 formulations. Both formulations had identical composition but differed in method of manufacturing. F8 dissolution decreased from 100.8 ± 1.3 to 81.9 ± 0.7% at 60 min compared to a 5.6% decrease in F9. This could be explained by a percentage increase in crystallinity to 40.1 and 11.9% in F8 and F9, respectively. Additionally, initial particle size and type of granulating fluid interplay had an impact on the solid-state of the drug and thus dissolution after storage. Initially, formulations manufactured by wet granulation had both amorphous and crystalline phases. However, the proportion of amorphous and crystalline phases was different in the granulated formulations depending on the particle size of the drug and granulating solvent used during manufacturing. Initially, the crystalline fraction was less if the particle size used was 32 µm in wet granulation. For example, the initial crystallinity of F3 and F8 formulations was 49.0 and 36.5% relative to the F10 formulation (directly compressed), respectively. F3 (75 µm), F8 (32 µm), and F10 (75 µm) formulation compositions were identical and differed in particle size and manufacturing methods. A significant increase in crystallinity was observed relative to their initial crystallinity after storage. An increase in crystallinity was 15.8 and 40.1% in the F3 and F8 formulations, respectively, due to higher initial amorphous phase content. A decrease in dissolution corresponding to a proportional increase in crystallinity was observed. Dissolution decreased by 8.0 and 18.9% at 60 min in F3 and F8 formulations, respectively. Dissolution changed from 38.5± to 21.9±% and 100.8± to 81.9±% in F3 and F8 formulations, respectively. A similar trend was observed in water–ethanol granulated formulations. However, a higher increase in crystallinity and a decrease in dissolution was observed due to higher solubility of the drug in the water–ethanol co-solvent, which resulted in a higher amorphous phase initially. For instance, F4 (75 µm) and F7 (32 µm) contained 54.3 and 50.7% crystalline fraction relative to F10. After storage, the crystalline fraction increased by 9.8 and 30.8% relative to the initial values in F4 and F7, respectively. Dissolution decreased from 37.6 ± 1.1 to 28.1 ± 1.9% and from 66.4 ± 1.4 to 45.3 ± 1.3% in F4 and F7, respectively. Absolute decrease in dissolution was 9.5 and 21.1% at 60 min in them.

To quantitatively compare the dissolution profiles before and after accelerated stability storage, the similarity factor (f_2_) was calculated using dissolution data collected at 5, 10, 20, 30, 45, and 60 min. Formulations F2–F5 exhibited similar dissolution profiles (f_2_ ≥ 50), whereas F1 and F6–F10 showed dissimilar profiles (f_2_ < 50). The lower f_2_ values were primarily attributable to greater reductions in drug release during the early stage of dissolution (10–30 min). The calculated f_2_ values, along with the corresponding dissolution data, are provided in Supplementary Tables S6 and S7.

## 3. Materials and Methods

### 3.1. Materials

Three major impurities (Digoxigenin bisdigitoxoside (DGB), digoxigenin monodigitoxoside (DGM), digoxigenin (DGG)) and DGX were acquired from Toronto Research Chemicals (Toronto, ON, Canada). Lactose monohydrate (LM) (SuperTab^®^14SD), lactose anhydrous (LA), microcrystalline cellulose, hydroxypropyl cellulose (HPC), and magnesium stearate were from DFE Pharma, Paramus, NJ, USA. Ethanol and methanol were obtained from Sigma-Aldrich (St. Louis, MO, USA). In-house Milli-Q Gradient A-10 (Millipore Corporation, Bedford, MA, USA) was used to obtain 18 MW deionized water, which was used in all experiments. All chemicals were of analytical grade and used directly as supplied.

### 3.2. Methods

#### 3.2.1. Experimental Design

A 2^3^ full factorial design to understand the impact of process and formulation variables on critical quality attributes of the DGX tablet using JMP 14 software (SAS, Cary, NC, USA). A formal risk assessment, such as Failure Mode and Effects Analysis (FMEA), was not performed in this study. However, the selection of the independent variables was guided by the physicochemical properties of the drug and excipients, including solubility, crystallinity, particle size, and other relevant material attributes, as well as the available literature and prior scientific knowledge. Independent variables studied were (X_1_) Lactose type (LM and LA), (X_2_) drug particle size (32 and 75 μm), and (X_3_) Granulating fluid (water and water–ethanol). Responses measured were crystallinity (%, Y_1_), disintegration time (DT) (min, Y_2_), percent drug dissolved at (Y_3_) 10, (Y_4_) 30, and (Y_5_) 60 min.

#### 3.2.2. Formulations

Eight DGX formulations (F1–F8) were formulated with different lactose types (anhydrous and monohydrate), microcrystalline cellulose, magnesium stearate, water, and ethanol using wet granulation methods, and two formulations (F9 and F10) with direct compression (Table 1). Two separate physical mixtures of the formulation were prepared, one containing LA (PM-1) and the other LM (PM-2). Tablets were compressed at compression forces of 3.0–5.0 kp in a rotary press with a 6 mm flat plain punch diameter (GlobePharma Inc., Monmouth Junction, NJ, USA). Before compression, 0.1% magnesium stearate was added as a lubricant. DGX Formulations were blended and compressed (approx. 250 tablets) and tested for hardness and mass variation. The hardness of the tablet was in the range (3.4–4.37 kp) on a Monsanto hardness tester. The mass of the tablet was 100 ± 10 mg.

#### 3.2.3. Hardness and Disintegration Time

The hardness of the tablet was measured using a tablet hardness tester (VK 200, Varian Inc., Cary, NC, USA). Disintegration tests were conducted on 6 tablets using USP disintegration (900 mL water medium at 37 ± 0.5 °C), and the time for the tablets to disintegrate completely was observed.

#### 3.2.4. Dissolution

Dissolution for the formulations was performed by the USP 1- Basket method in 500 mL of water at 120 rpm and 37 °C. Samples were collected at different time points such as 5, 10, 20, 30, 45, and 60 min, and the amount of drug dissolved from the tablet was quantified by injecting 50 μL of the sample into the UPLC system. The dissolution studies were conducted in triplicate for each formulation [31,45,46]. The dissolution method used in this study was previously developed and validated as a discriminatory method for digoxin formulations. Appropriate sink conditions and the discriminatory ability of the method have been established and reported in our previous study [29].

#### 3.2.5. X-Ray Powder Diffraction

XRPD data were collected using a Bruker D2 Phaser SSD 160 diffractometer (Bruker AXS, Madison, WI, USA) equipped with a LYNXEYE scintillation detector, and Cu Kα radiation (λ = 1.54184 Ǻ) was operated at 30 KV and 10 mA. Approximately 650 mg of powder was placed into the sample holder. Two methods were used to collect X-ray powder diffraction (XPRD) data of the formulation. A diffractogram was collected over the 2θ range of 13–20° in the region where prominent drug reflection bands were observed, with a step size of 0.0202° at 60 s exposure time per step (396 total steps, total time: 11 h). Samples were rotated at 15 rpm during the measurement to obtain an average diffractogram [29].

#### 3.2.6. Assay

Assay analysis was performed in five replicates. One tablet was placed in a 50 mL volumetric flask containing water and methanol (50:50% *v*/*v*). The flasks were sonicated at 25 °C for 20 min until the tablets completely disintegrated. The volume was adjusted with methanol, and the solution was filtered through 0.45 µm syringe filters. A 50 µL sample was injected into the UPLC system for the assay determination [47].

#### 3.2.7. Stability Study

Stability studies were performed under accelerated conditions (40 °C/75% RH) in accordance with the ICH guidelines. The products were stored in an HDPE bottle at 40 °C/75% RH for 30 days. Physical and chemical parameters of the tablets were evaluated at the beginning and after 30 days [48].

#### 3.2.8. Ultra-Performance Liquid Chromatography

A common analytical method was developed for the drug and impurities (DGG, DGM, and DGB). The system consisted of quaternary pumps, an autosampler, and a diode array detector set at a 220 nm wavelength. The column temperature maintained for the assay, impurity, and dissolution was 40 °C. Assay, impurity and dissolution were separated on a Luna^®^ C8 column (4.6 × 150 mm, 3 µm packing) with a C8 guard column (Security Guard™; Phenomenex, Torrance, CA, USA). The mobile phase was 73% Water and 27% acetonitrile, flowing isocratically at a 0.6 mL/min flow rate, and the run time was 25 min [29,49]. Representative chromatograms demonstrating the specificity of the developed UPLC method are provided in the Supplementary Data. The calibration curves for digoxigenin, monodigitoxoside, bisdigitoxoside, and digoxin are in the Supplementary Data, confirming the excellent linearity of the method.

#### 3.2.9. Statistical Analysis

Data were presented as mean standard deviation. Statistical significance was determined using the *p*-value. *p* < 0.05 was deemed statistically significant, while *p* > 0.05 was deemed statistically insignificant. The complete ANOVA results are presented in Supplementary Table S5.

## 4. Conclusions

NTI drugs, such as DGX, are sensitive to minor alterations in product quality, which may either result in inconsistency in clinical outcome or enhance the toxicity. Therefore, there is a need for a thorough understanding of how formulation excipients and process variables affect the stability of the drug. Assay values of the formulations did not change significantly (*p* > 0.05) compared to initial values after storage. No impurities or new peaks were detected in the stability samples, indicating either no drug degradation or that the degradation products were below the limit of detection of the method (31.5–44.6 ng/mL). Drug particle size significantly influenced dissolution, and lactose type affected the DT. In direct compression formulations, a decrease in dissolution rate, especially at 10 and 20 min, was higher than at 60 min, which may be related to an increase in hardness and DT values. However, a decrease in dissolution of 5.6–8.7% at 60 min was observed after storage from the initial values, which may be due to an increase in crystallinity. Wet granulation resulted in partial amorphization of the drug, particularly in water–ethanol systems, leading to enhanced initial dissolution but reduced stability due to recrystallization during storage. Short-term stability studies indicated that moisture-induced microstructural changes increased hardness and DT, which contributed to reduced dissolution performance. However, long-term stability studies (25 °C/60% RH) and evaluations under patient in-use conditions are needed to determine whether these changes are relevant under those storage conditions. An increased crystallinity after storage, compared to the initial crystallinity of wet granulated formulations.

## Figures and Tables

**Table 1:** Formulations with process variables.

Formulations	Weight (mg/Tablet)							Process Variables	
	Digoxin	Lactose Monohydrate	Lactose Anhydrous	Microcrystalline Cellulose	Hydroxypropyl Cellulose	Magnesium Stearate	Total Tablet Weight (mg)	Digoxin Particle Size (µm)	Granulating Solvent
F1	0.25	60	0	35.75	3	1	100	32	Ethanol
F2	0.25	60	0	35.75	3	1	100	75	Ethanol
F3	0.25	0	60	35.75	3	1	100	75	Ethanol
F4	0.25	0	60	35.75	3	1	100	75	Water–Ethanol
F5	0.25	60	0	35.75	3	1	100	75	Water–Ethanol
F6	0.25	60	0	35.75	3	1	100	32	Water–Ethanol
F7	0.25	0	60	35.75	3	1	100	32	Water–Ethanol
F8	0.25	0	60	35.75	3	1	100	32	Ethanol
F9	0.25	0	60	35.75	3	1	100	32	-
F10	0.25	0	60	35.75	3	1	100	75	-

## Data Availability

The data presented in this study are available from the corresponding author upon reasonable request.

## Supplementary Materials

The following supporting information can be downloaded at: https://www.mdpi.com/article/10.3390/ph19081185/s1, Figure S1: Chromatograms of the mobile phase, dissolution medium, placebo, and digoxin tablet in 0.1 N HCl medium; Figure S2: Calibration curves of (A) digoxigenin, (B) monodigitoxoside, (C) bisdigitoxoside, and (D) digoxin; Table S1: UPLC method validation data of digoxin; Table S2: UPLC method validation data of digoxigenin; Table S3: UPLC method validation data of digoxigenin monodigitoxoside; Table S4: UPLC method validation data of digoxigenin bisdigitoxoside; Table S5: Parameter estimates of formulation factors for the full factorial design; Table S6: Difference factor (f_1_) and similarity factor (f_2_) values comparing the dissolution profiles of formulations F1–F10 before and after accelerated stability storage (40 °C/75% RH); Table S7: Percentage changes in dissolution (%) at the early dissolution stage (10, 20, and 30 min) between initial and stability samples of formulations F1–F10.
