# Supplementary material for: Effect of Formulation and Process Variables on the Stability and Quality Attributes of Digoxin Formulations
Source: Pharmaceuticals (Basel). 2026 Jul 29;19(8):1185. doi: 10.3390/ph19081185 (PMC13516292; doi:10.3390/ph19081185)
Supplement: Supplementary file 1 [file pharmaceuticals-19-01185-s001.zip › pharmaceuticals-4419616-supplementary.pdf]

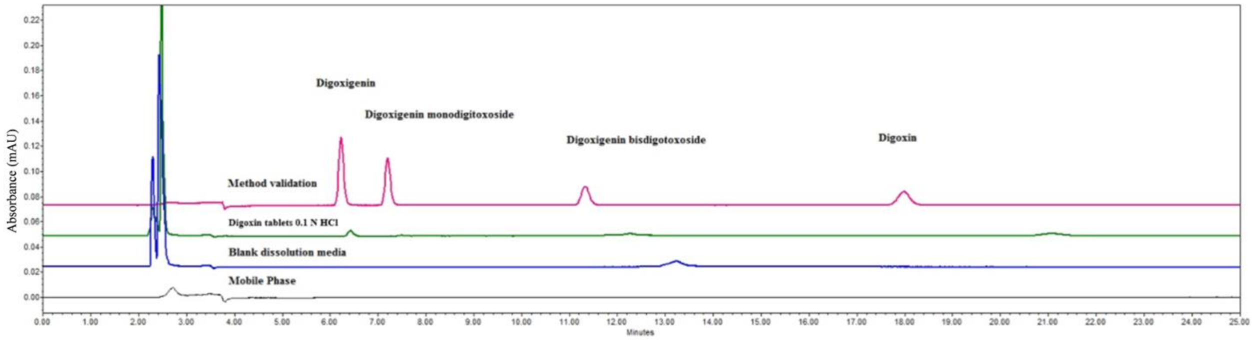

Figure S1: Chromatograms of the mobile phase, dissolution medium, placebo, and digoxin tablet in 0.1 N HCl medium.

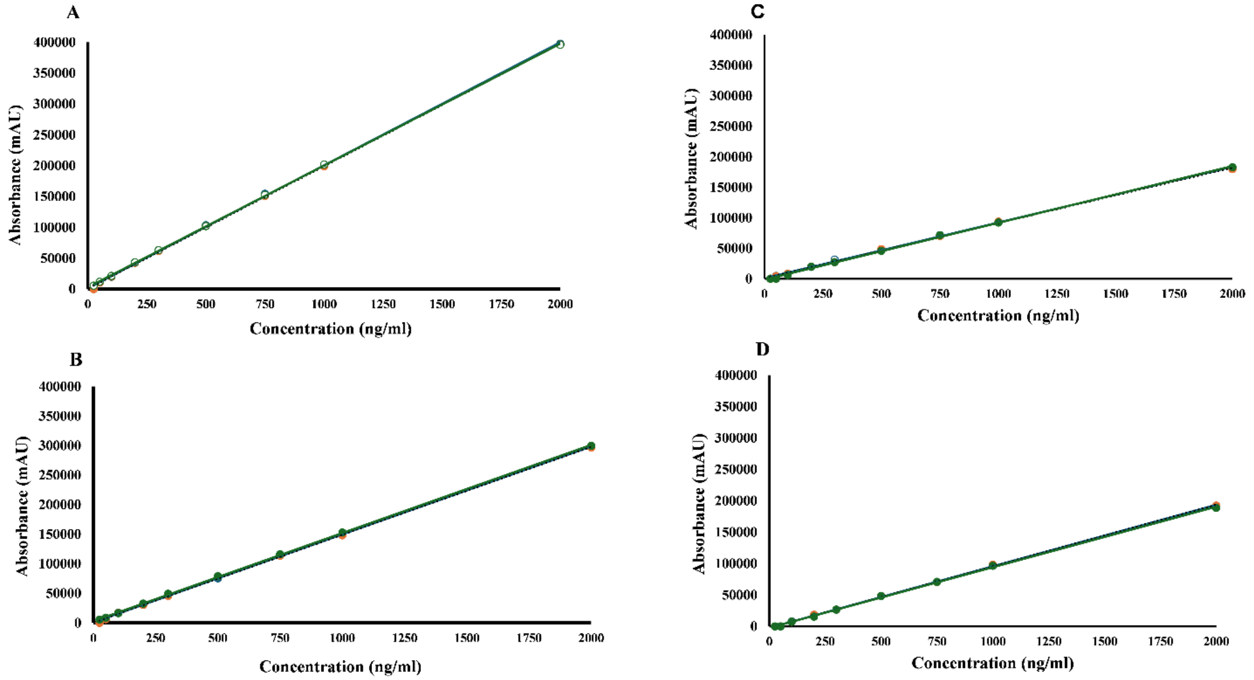

Figure S2: Calibration curves of (A) digoxigenin, (B) monodigitoxoside, (C) bisdigitoxoside, (D) digoxin.

Table S1. UPLC method validation data of Digoxin.

Parameter	Day 1	Day 2	Day 3	Mean ± SD
System suitability				
Retention time (% RSD) (≤2)	0.05	0.04	0.05	0.04±0.0
Area (% RSD) (≤2)	0.84	0.93	0.72	0.83±0.1
Plates (column) (>3000)	23558	235858	24741	94719±12
USP tailing (<1.5)	1.03	1.04	1.03	1.03±0.0
Linearity (correlation coefficient, r2)				
Digoxin	0.999	0.999	0.999	0.999±0.0
Slope				
Digoxin	97.991	96.511	96.418	96.9±0.8
y-Intercept				
Digoxin	-2342.4	1099	-2342.4	1195.2 ±19
Analytical range (25-2000 ng/ml)				
Calibrators	8	8	8	8
LOD (ng/ml)	25	25	25	25
LOQ (ng/ml)	100	100	100	100
Accuracy (n=6, % Recovery)				
100 ng/ml	100.1	98.3	99.5	99.3 ± 0.9
500 ng/ml	99.2	102.1	100.2	100.5 ± 1.4
1000 ng/ml	102.8	107.8	104.6	103.7 ± 0.9
Precision (n=6, % RSD)				
100 ng/ml	3.0	3.1	3.3	3.2±0.1
500 ng/ml	3.7	3.1	3.9	3.6±0.0
1000 ng/ml	0.6	0.4	0.1	0.4±0.0

Table S2. UPLC method validation data of digoxigenin.

Parameter	Day 1	Day 2	Day 3	Mean ± SD
System suitability				
Retention time (% RSD) (≤2)	0.04	0.01	0.03	0.02±0.0
Area (% RSD) (≤2)	0.84	0.93	0.72	0.83±0.1
Plates (column) (>3000)	23558	235858	24741	94719±12
USP tailing (<1.5)	1.03	1.04	1.03	1.03±0.0
Linearity				
Correlation coefficient	0.999	0.999	0.999	0.999±0.0
Slope	198.9	96.511	96.418	96.9±0.8
y-Intercept	1432.5	1099	-2342.4	1195.2 ±19
Limit of detection (ng/ml)	6	6	6	6
Limit of quantification (ng/ml)	35	35	35	35
Accuracy (n=6, % Recovery)				
100 ng/ml	101.4	100.3	101.6	101.1 ± 0.7
500 ng/ml	100.9	102.2	99.1	100.7 ± 1.5
1000 ng/ml	101.8	103.8	103.6	103.0 ± 1.1
Precision (n=6, % RSD)				
100 ng/ml	0.1	0.4	0.3	0.2±0.1
500 ng/ml	1.2	1.4	1.9	1.5±0.3
1000 ng/ml	2.6	1.8	1.7	2.0±0.4

Table S3. UPLC method validation data of digoxigenin monodigitoxoside

Parameter	Day 1	Day 2	Day 3	Mean ± SD
System suitability				
Retention time (% RSD) (≤2)	0.03	0.02	0.03	0.02±0.0
Area (% RSD) (≤2)	0.84	0.93	0.72	0.83±0.1
Plates (column) (>3000)	23558	235858	24741	94719±12
USP tailing (<1.5)	1.03	1.04	1.03	1.03±0.0
Linearity				
Correlation coefficient	0.999	0.999	0.999	0.999±0.0
Slope	148.73	148.68	149.1	148.83±0.2
y-Intercept	901.44	510.56	2872.6	1428.2 ±1266
Limit of detection (ng/ml)	9	9	9	9
Limit of quantification (ng/ml)	40	40	40	40
Accuracy (n=6, % Recovery)				
100 ng/ml	100.1	98.3	99.5	99.3 ± 0.9
500 ng/ml	99.2	102.1	100.2	100.5 ± 1.4
1000 ng/ml	102.8	103.8	104.6	103.7 ± 0.9
Precision (n=6, % RSD)				
100 ng/ml	0.1	0.1	0.4	0.2±0.1
500 ng/ml	1.5	1.4	1.3	1.4±0.1
1000 ng/ml	2.6	2.2	2.7	2.5±0.2

Table S4. UPLC method validation data of digoxigenin bisdigitoxoside

Parameter	Day 1	Day 2	Day 3	Mean ± SD
System suitability				
Retention time (% RSD) (≤2)	0.02	0.02	0.01	0.02±0.0
Area (% RSD) (≤2)	0.84	0.93	0.72	0.83±0.1
Plates (column) (>3000)	23558	235858	24741	94719±12
USP tailing (<1.5)	1.03	1.04	1.03	1.03±0.0
Linearity				
Correlation coefficient	0.999	0.999	0.999	0.999±0.0
Slope	91.316	90.751	93.325	91.79±1.3
y-Intercept	975.27	635.83	647.2	752.76 ±192.7
Limit of detection (ng/ml)	11	11	11	11
Limit of quantification (ng/ml)	39	39	39	39
Accuracy (n=6, % Recovery)				
100 ng/ml	100.1	98.3	99.5	99.3 ± 0.9
500 ng/ml	99.2	102.1	100.2	100.5 ± 1.4
1000 ng/ml	102.8	103.8	104.6	103.7 ± 0.9
Precision (n=6, % RSD)				
100 ng/ml	2.4	2.3	2.1	0.2±0.1
500 ng/ml	3.1	2.9	2.3	1.4±0.1
1000 ng/ml	3.6	3.2	3.5	2.5±0.2

Table S5. Parameter estimates of formulation factors for the full factorial design

Response	Factor	Estimate	Std. Error	t Ratio	*p*-value	Significance
**Hardness**	Lactose	0.7125	0.2200	3.24	0.1907	NS
	Particle Size	0.7000	0.2200	3.18	0.1939	NS
	Solvent	-0.0075	0.2200	-0.03	0.9783	NS
	Lactose × Particle Size	0.2825	0.2200	1.28	0.4212	NS
	Lactose × Solvent	-0.2000	0.2200	-0.91	0.5303	NS
	Particle Size × Solvent	-0.2625	0.2200	-1.19	0.4441	NS
**Disintegration**	Lactose	2.8388	0.8838	3.21	0.1921	NS
	Particle Size	1.3388	0.8838	1.51	0.3714	NS
	Solvent	0.0913	0.8838	0.10	0.9345	NS
	Lactose × Particle Size	1.3563	0.8838	1.53	0.3677	NS
	Lactose × Solvent	0.0988	0.8838	0.11	0.9292	NS
	Particle Size × Solvent	-0.9163	0.8838	-1.04	0.4885	NS
**Dissolution T10**	Lactose	-0.3625	0.3875	-0.94	0.5212	NS
	**Particle Size**	**-6.3625**	**0.3875**	**-16.42**	**0.0387**	**Significant**
	Solvent	-3.0625	0.3875	-7.90	0.0801	NS
	Lactose × Particle Size	-0.4125	0.3875	-1.06	0.4801	NS
	Lactose × Solvent	0.8875	0.3875	2.29	0.2621	NS
	Particle Size × Solvent	2.2375	0.3875	5.77	0.1092	NS
**Dissolution T30**	Lactose	-1.2000	0.4750	-2.53	0.2399	NS
	**Particle Size**	**-21.8000**	**0.4750**	**-45.89**	**0.0139**	**Significant**
	**Solvent**	**-10.7250**	**0.4750**	**-22.58**	**0.0282**	**Significant**
	Lactose × Particle Size	-1.4000	0.4750	-2.95	0.2082	NS
	Lactose × Solvent	3.9250	0.4750	8.26	0.0767	NS
	**Particle Size × Solvent**	**9.6250**	**0.4750**	**20.26**	**0.0314**	**Significant**
**Dissolution T60**	Lactose	-1.9000	2.3750	-0.80	0.5704	NS
	Particle Size	-22.5250	2.3750	-9.48	0.0669	NS
	Solvent	-9.0000	2.3750	-3.79	0.1643	NS
	Lactose × Particle Size	0.3750	2.3750	0.16	0.9003	NS
	Lactose × Solvent	-0.0500	2.3750	-0.02	0.9866	NS
	Particle Size × Solvent	10.0750	2.3750	4.24	0.1474	NS
**Relative % Intensity**	Lactose	-0.2750	0.4450	-0.62	0.6476	NS
	Particle Size	1.9600	0.4450	4.40	0.1421	NS
	Solvent	1.1300	0.4450	2.54	0.2388	NS
	Lactose × Particle Size	1.9825	0.4450	4.46	0.1406	NS
	Lactose × Solvent	3.6475	0.4450	8.20	0.0773	NS
	Particle Size × Solvent	-1.7425	0.4450	-3.92	0.1592	NS

Table S6. Difference factor (f_1) and Similarity factor (f_2) values comparing the dissolution profiles of formulations F1–F10 before and after accelerated stability storage (40°C/75% RH).

**Formulation**	**Difference factor (f_1)**	**Similarity factor (f_2)**	**Interpretation**
F1	36.22	30.06	Not similar
F2	31.00	57.73	Similar
F3	34.00	64.34	Similar
F4	34.20	60.69	Similar
F5	21.47	61.89	Similar
F6	49.34	37.44	Not similar
F7	31.87	42.23	Not similar
F8	39.15	29.01	Not similar
F9	22.70	31.76	Not similar
F10	25.07	37.36	Not similar

Similarity factor (f_2) was calculated according to FDA/EMA recommendations using the dissolution data at 5, 10, 20, 30, 45, and 60 min. Dissolution profiles with $f_2 \geq 50$ were considered similar, whereas $f_2 < 50$ indicated significant differences.

Table S7. Percentage changes in dissolution (%) at the early dissolution stage (10, 20, and 30 min) between initial and stability samples of formulations F1–F10.

**Formulation**	**T10 min**	**T20 min**	**T30 min**
F1	10.3	25.8	36.8
F2	3.5	8.9	10.2
F3	1.6	4.0	5.1
F4	3.0	7.5	7.0
F5	0.5	1.1	0.9
F6	5.7	14.3	15.4
F7	2.8	10.0	16.0
F8	13.6	34.0	38.4
F9	19.8	52.7	1.9
F10	15.0	37.8	11.3
